# Neuromuscular electrical stimulation enhances neuromuscular activation and motor performance in badminton players during forward lunge footwork

**DOI:** 10.3389/fphys.2026.1880726

**Published:** 2026-06-10

**Authors:** Xuerui Li, Yi Sheng

**Affiliations:** 1 College of Physical Education, Chongqing University, Chongqing, China; 2 Shanghai University of Sport, Shanghai, China

**Keywords:** badminton, co-activation index, forward lunge footwork, intermuscular coherence coupling, intermuscular time–frequency coherence model, muscle activation, neuromuscular electrical stimulation, surface electromyography

## Abstract

**Background:**

Forward lunge footwork is a key badminton-specific movement requiring rapid initiation, landing control, postural stability, and push-off recovery. This study examined whether pre-competition neuromuscular electrical stimulation (NMES) combined with weighted squats improves forward lunge performance and neuromuscular control in badminton players.

**Methods:**

Thirty-six male badminton players were randomly assigned to three groups: weighted squats, fake stimulation combined with weighted squats, and real NMES combined with weighted squats. All participants completed the assigned pre-activation intervention followed by six valid badminton-specific star-shuttle trials. Forward lunge performance was assessed using total completion time. Surface electromyography was recorded from 14 right-side muscles, and high-speed video was used to identify the forward lunge movement cycle. Integrated EMG, co-activation index, and intermuscular time-frequency coherence were calculated. Between-group differences were analyzed using one-way ANOVA with correction for multiple comparisons.

**Results:**

The Real Stimulation group showed significantly shorter completion time than the Weighted Squats and Fake Stimulation groups. Compared with the other two groups, the Real Stimulation group also demonstrated higher iEMG in GLM, VL, VM, LD, and ABS, as well as greater co-activation of VL–BF and GLM–BF. Intermuscular time-frequency coherence was also enhanced in selected muscle pairs across α, β, and γ frequency bands, indicating stronger task-specific neuromuscular coupling during the forward lunge cycle.

**Conclusion:**

Pre-competition NMES combined with weighted squats improved badminton-specific forward lunge performance. This improvement was accompanied by increased activation of key lower-limb and trunk-related muscles, enhanced lower-limb co-activation, and stronger frequency-specific intermuscular coherence. These findings suggest that NMES may be an effective adjunct strategy for acute badminton pre-activation.

## Introduction

Badminton is a high-speed racket sport characterized by rapid attack–defense transitions, frequent changes of direction, explosive acceleration and deceleration, and continuous adjustment of body position. During competition, players are required to move quickly to the optimal hitting position, stabilize the body under time pressure, and return efficiently for the next technical action. Therefore, footwork is not only the basis of court movement but also an important determinant of stroke quality, tactical execution, and competitive performance. Among various badminton-specific footwork patterns, Forward Lunge footwork is one of the most representative and frequently used movement patterns. It is widely involved in front-court interception, net play, receiving drop shots, defensive recovery, and transition from defense to attack. The Forward Lunge movement requires rapid lower-limb initiation, accurate stepping, landing and braking, support stabilization, and push-off recovery. At the same time, it also depends on trunk postural control and upper-limb coordination to maintain balance and prepare for subsequent technical actions. Therefore, Forward Lunge footwork provides an important functional task for evaluating badminton players’ movement performance and neuromuscular control during sport-specific dynamic actions (Lam et al., 2020).

Pre-competition activation is an essential component of athletic preparation. Its primary purpose is to optimize the physiological and neuromuscular state before competition, thereby improving the immediate output capacity of subsequent sport-specific movements. Relevant research evidence has shown that properly designed pre-activation strategies can increase neural excitability, enhance motor unit recruitment, improve muscle contractile readiness, and promote explosive movement performance (Tillin and Bishop, 2009). In many sports, short-duration resistance exercise, dynamic warm-up, and sport-specific activation have been shown to improve sprinting, jumping, change-of-direction ability, and rapid movement performance (Behm and Chaouachi, 2011). For badminton players, pre-competition activation is particularly important because court movement requires not only speed but also precise control of the center of mass, braking stability, and rapid recovery after lunging. If the pre-activation strategy is insufficient, players may fail to reach the optimal neuromuscular state, whereas excessive activation may induce fatigue and reduce subsequent movement efficiency. Therefore, developing an effective pre-competition activation strategy that enhances lower-limb power, postural stability, and intermuscular coordination without producing excessive fatigue is highly relevant for badminton-specific performance enhancement (Phomsoupha and Laffaye, 2015).

Weighted squats are commonly used as a lower-limb pre-activation exercise because they can effectively stimulate the quadriceps femoris, gluteal muscles, hamstrings, and calf muscles. These muscles are highly involved in lower-limb extension, closed-chain support, landing control, and push-off force production. The biomechanical characteristics of Forward Lunge footwork are closely related to these functions. During the Forward Lunge cycle, the athlete must initiate movement from the central ready position, complete a rapid anterior lunge, control the body during landing and braking, and then push off to return to the starting position. This process requires coordinated hip, knee, and ankle joint actions, as well as stable lower-limb support. Therefore, weighted squats can serve as a practical and ecologically valid foundation for badminton-specific pre-activation (Schoenfeld, 2010). However, the acute effect of weighted-squat activation alone may be limited by individual training status, fatigue tolerance, and variability in neural readiness. Although resistance-based pre-activation may enhance subsequent performance through post-activation performance enhancement, its effect is not always stable in high-speed sport-specific movement tasks (Seitz and Haff, 2016). Accordingly, combining weighted squats with an additional neuromuscular modulation strategy may provide a more targeted approach to improving the immediate readiness of the neuromuscular system.

Neuromuscular electrical stimulation is an external stimulation method that directly acts on the peripheral neuromuscular system. By delivering electrical pulses to target muscles, NMES can induce muscle contractions, increase local neuromuscular excitability, and facilitate motor unit recruitment. Relevant research evidence suggests that NMES may improve muscle activation, explosive output, and neuromuscular control when applied under appropriate stimulation parameters (Bickel et al., 2011). In the context of Forward Lunge footwork, applying NMES to the bilateral quadriceps femoris during pre-activation may enhance knee extension readiness, lower-limb support capacity, landing stability, and push-off recovery. These effects may contribute to faster completion of the badminton-specific footwork task. However, improvement in movement performance cannot be fully explained by the activation of a single muscle. Forward Lunge footwork is a complex whole-body movement that requires coordinated interactions among lower-limb, trunk, and upper-limb muscles. Therefore, it is necessary to examine not only muscle activation level but also intermuscular coordination and neural coupling characteristics. Intermuscular time–frequency coherence provides a useful analytical approach for quantifying synchronization between muscle pairs in specific time and frequency domains. Coherence in the α, β, and γ frequency bands has been associated with different aspects of motor control, including rhythmic stabilization, corticospinal drive, and rapid neural regulation during high-intensity or fast movements (Laine and Valero-Cuevas, 2017). Thus, combining performance outcomes with intermuscular time–frequency coherence analysis may help clarify the neuromuscular mechanism by which NMES enhances Forward Lunge footwork performance.

Recent developments in neuromusculoskeletal research have emphasized the importance of advanced modeling approaches for evaluating dynamic functional performance. Traditional performance indicators, such as completion time, speed, or force output, are useful for describing external movement results, but they are insufficient for explaining the underlying neuromuscular control strategies during complex sport-specific tasks. In lower-extremity dynamic actions, performance deficits or improvements often involve changes in muscle activation, antagonist–agonist co-activation, modular muscle coordination, and frequency-specific intermuscular coupling (Hug, 2011). Surface electromyography-based models, including integrated EMG analysis, co-activation index, non-negative matrix factorization, and intermuscular time–frequency coherence, can provide a more comprehensive description of neuromuscular control during functional movement. Such analytical frameworks are consistent with the current direction of precision neuromusculoskeletal assessment, which aims to integrate biomechanical and neuromuscular data to evaluate movement function, optimize exercise performance, and guide individualized intervention strategies (Lloyd and Besier, 2003). Although badminton athletes do not represent a clinical disorder population, their high-speed Forward Lunge footwork imposes substantial demands on lower-extremity neuromuscular control, dynamic stability, and whole-body coordination. Therefore, this sport-specific task provides a meaningful model for investigating how acute neuromuscular intervention influences lower-extremity movement performance and intermuscular control.

Therefore, the purpose of this study was to investigate the acute effects of pre-competition neuromuscular electrical stimulation on neuromuscular activation and Forward Lunge footwork performance in badminton players, using an intermuscular time–frequency coherence model. Specifically, this study compared three pre-activation strategies: weighted squats, fake stimulation combined with weighted squats, and real NMES combined with weighted squats. Forward Lunge footwork performance was assessed using the total completion time of a badminton-specific star-shuttle test, while neuromuscular control was evaluated using surface electromyography-derived indicators, including integrated EMG, co-activation index, muscle synergy parameters, intermuscular time–frequency coherence, and the area of significant coherence. It was hypothesized that, compared with weighted squats and fake stimulation, real NMES would improve Forward Lunge footwork performance, increase the activation of key lower-limb, trunk, and upper-limb muscles, enhance functional muscle-pair co-activation, and strengthen intermuscular coherence in specific α, β, and γ frequency bands. The findings of this study may provide evidence for optimizing badminton pre-competition activation strategies and offer a neuromuscular modeling framework for understanding the mechanisms underlying sport-specific lower-limb performance enhancement.

## 2 Participants and methods

### 2.1 Participants

An *a priori* sample size calculation was performed using G*Power software. Because the primary aim of the study was to compare the acute effects of three pre-activation conditions on Forward Lunge Test performance, the sample size calculation was based on the primary outcome, namely Forward Lunge Test completion time. The test family was set to F tests, and ANOVA: Fixed effects, omnibus, one-way was selected as the statistical model. The parameters were set as follows: assumed effect size f = 0.60, significance level α = 0.05, statistical power = 0.80, and number of groups = 3 (Faul et al., 2007). The assumed moderate-to-large effect size was selected based on previous evidence showing meaningful acute effects of post-activation performance enhancement and neuromuscular electrical stimulation on explosive or sport-specific movement performance, as well as the expected effect of NMES combined with weighted squats on badminton-specific Forward Lunge performance.

The calculation indicated that a minimum total sample size of 30 participants was required. Considering potential participant withdrawal, invalid test trials, poor-quality surface electromyography signals, or synchronization errors during data collection, 36 male badminton players were ultimately recruited, with 12 participants in each group. The study was powered for the primary performance outcome. The EMG-derived outcomes, including iEMG, co-activation index, and intermuscular time-frequency coherence, were treated as secondary neuromuscular outcomes and were not powered separately. Therefore, these secondary findings were interpreted as supportive mechanistic evidence for the performance results.

A total of 36 male badminton players were included in this study. All participants had systematic badminton-specific training experience and had achieved a top-three ranking in provincial-level badminton competitions or demonstrated an equivalent competitive level. After completing basic information registration and screening according to the inclusion and exclusion criteria, participants were randomly allocated to the Weighted Squats, Fake Stimulation, and Real Stimulation groups, with 12 participants in each group. All three groups completed the same multi-directional star-shuttle test, surface electromyography recording, and high-speed video recording procedures; the only difference among groups was the type of acute intervention. Participant characteristics are presented in **Table 1**.

**Table 1:** Participant characteristics (M ± SD, N = 36).

Variable	Weighted squats	Fake stimulation	Real stimulation	F	P
Age (years)	20.17 ± 1.34	21.58 ± 1.67	21.92 ± 1.83	2.96	0.066
Height (cm)	176.42 ± 4.85	177.08 ± 5.12	176.75 ± 4.68	0.05	0.948
Body mass (kg)	68.35 ± 5.74	69.12 ± 6.21	68.87 ± 5.96	0.05	0.952
Training age (years)	8.25 ± 1.60	8.58 ± 1.73	8.42 ± 1.51	0.12	0.889
Dominant limb, right/left	12/0	12/0	12/0	—	—
Baseline completion time (s)	10.91 ± 0.36	10.86 ± 0.39	10.88 ± 0.34	0.06	0.942

Values are presented as mean ± standard deviation. Age, height, body mass, training age, and baseline completion time were compared using one-way ANOVA. Baseline completion time refers to the pre-intervention performance in the Forward Lunge Test. Dominant limb distribution was not statistically compared because all participants were right-limb dominant. No significant between-group differences were observed in any baseline characteristic or baseline Forward Lunge Test performance variable.

Participants were eligible if they were male badminton players aged 18–26 years, had systematic badminton-specific training experience, had achieved a top-three ranking in provincial-level competitions or an equivalent competitive level, and had no neurological, musculoskeletal, or cardiovascular conditions affecting exercise performance. Participants were excluded if they had any contraindication to neuromuscular electrical stimulation, unresolved lower-limb or trunk injury, high-intensity lower-limb training or competition within 48 h before testing, inability to complete the weighted squat or star-shuttle test correctly, or unusable data due to equipment failure or poor signal quality. All participants provided written informed consent before testing. The study was approved by the Ethics Committee of Shanghai University of Sport and was conducted in accordance with ethical principles for human-subject research (No.102772024RT064).

### 2.2 Instruments

#### 2.2.1 Timing system for the star-shuttle test

**Figure 1:**
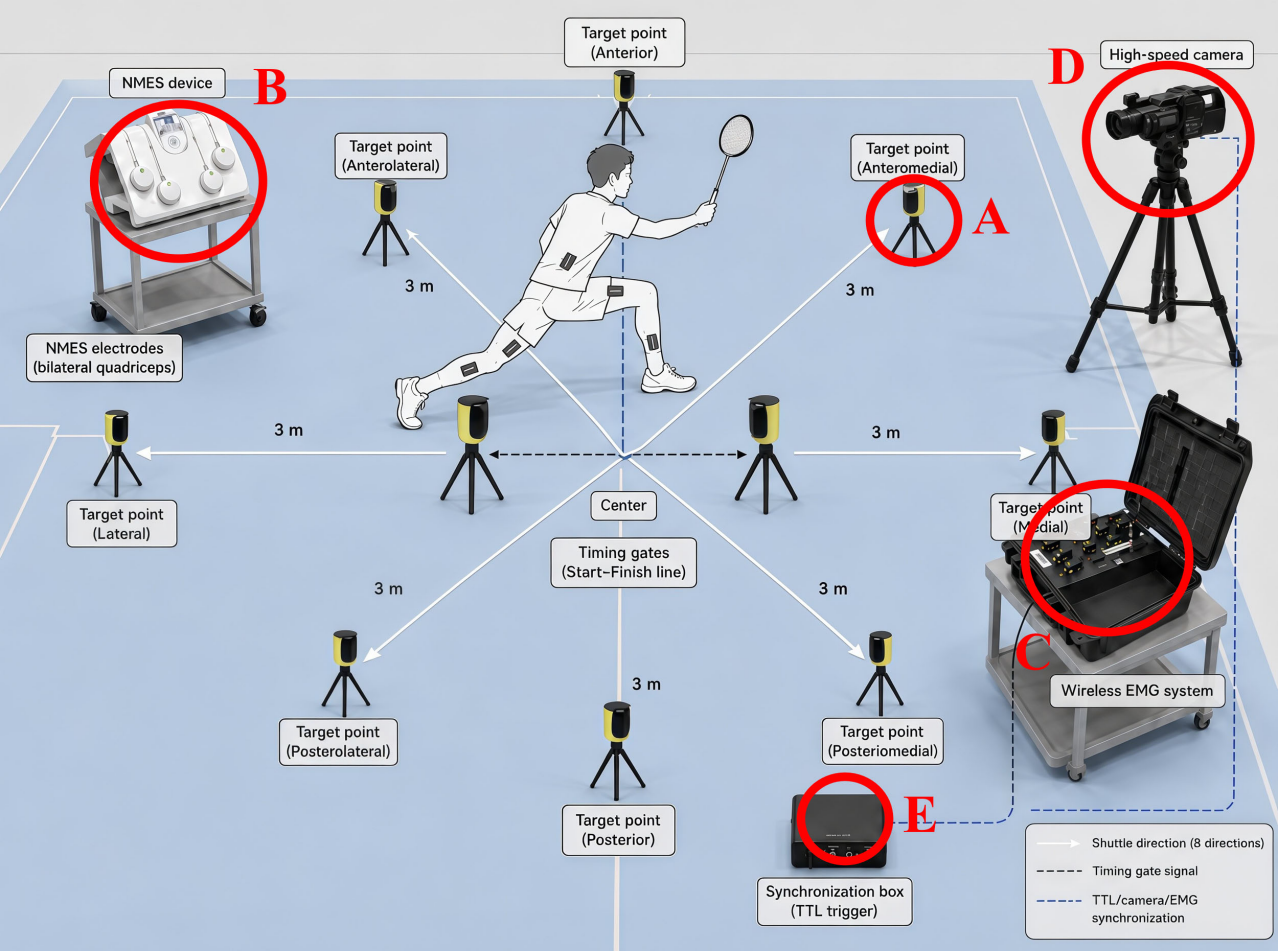
Schematic illustration of the experimental equipment and on-site setup. **A** is a speed measurement device, **B** is a neuromuscular electrical stimulation device, **C** is a surface electromyography device, **D** is a high-speed camera, and **E** is a synchronization device.

Forward Lunge footwork performance was assessed using a Microgate Witty Timing System (Microgate, Bolzano, Italy). The system consisted of wireless photocell timing gates, reflectors, a timing unit, and data management software. The photocell gates were configured in start–stop mode to record the total completion time of the star-shuttle test. The timing gates were positioned at the start–finish line of the test area. Participants started from the central position, completed the prescribed multidirectional star-shuttle route, and returned to the start–finish line after all target directions had been completed. The system automatically recorded the total time from the initial triggering of the start gate to the final triggering of the finish gate. Only the total completion time was extracted as the performance outcome; split times were not analyzed. During each trial, surface electromyography and high-speed video data were collected synchronously. The timing system was used to quantify overall movement performance, whereas the high-speed video system was used to identify the onset and offset of the Forward Lunge movement cycle, and the surface electromyography system was used to record muscle activity during the selected cycle (**Figure 1A**).

#### 2.2.2 Neuromuscular electrical stimulation device

Neuromuscular electrical stimulation was delivered using a Compex SP 8.0 stimulator (Compex/DJO, USA). The device was used for the Fake Stimulation and Real Stimulation conditions, whereas the Weighted Squats group did not receive electrical stimulation. Self-adhesive electrodes measuring 5 cm × 5 cm were placed bilaterally over the quadriceps femoris, primarily covering the rectus femoris and vastus medialis regions. In the Real Stimulation condition, stimulation frequency was set at 75 Hz, pulse width at 400 μs, and the on–off duty cycle at 6 s:12 s. Stimulation intensity was adjusted to 80%–90% of the individual maximal tolerable intensity to elicit visible and tolerable muscle contractions. In the Fake Stimulation condition, electrode placement, device connection, and operating procedures were identical to those used in the Real Stimulation condition; however, stimulation intensity was maintained at or below the sensory threshold, producing only mild cutaneous sensation without visible muscle contraction (**Figure 1B**).

#### 2.2.3 Surface electromyography system

Surface electromyographic signals were recorded using a Delsys Trigno Wireless EMG System (Delsys Inc., USA). The system was used to collect multichannel wireless electromyographic data from the target muscles during the Forward Lunge movement cycle within the star-shuttle test. The sampling frequency was set at 2000 Hz, and the signal bandwidth was set at 20–450 Hz. Before electrode placement, the skin over each target muscle was prepared by shaving when necessary, cleaning with alcohol, and allowing the skin to dry. Electrodes were placed over the muscle belly and aligned parallel to the presumed muscle fiber direction. Additional fixation was applied using medical tape or elastic film to minimize electrode displacement and motion artefacts during rapid acceleration, lunge, braking, and recovery phases (**Figure 1C**).

#### 2.2.4 High-speed video system

Movement events were recorded using a Photron FASTCAM Mini AX200 high-speed camera system (Photron, Japan). The sampling frequency was set at 200 Hz. The camera was positioned to capture the central starting position, the Forward Lunge movement path, and the return-to-center phase throughout the star-shuttle test. The high-speed video system was not used to calculate total test completion time. Instead, it was used for offline frame-by-frame identification of the onset and offset of the Forward Lunge movement cycle, which enabled the corresponding surface electromyography signals to be extracted for subsequent analysis (**Figure 1D**).

#### 2.2.5 Synchronization system

A TTL trigger was used to synchronize the timing system, surface electromyography system, and high-speed video system. Before testing, all systems were connected through external trigger interfaces and configured to use a common trigger signal. At the beginning of each trial, the trigger signal simultaneously initiated timing, electromyographic recording, and high-speed video acquisition, thereby establishing a shared temporal reference across systems. During offline processing, the TTL trigger was used as the common time reference. The timing system provided the total completion time of the star-shuttle test, the high-speed video system identified the Forward Lunge movement cycle, and the surface electromyography data corresponding to that cycle were extracted for neuromuscular analysis (**Figure 1E**).

### 2.3 Experimental design

Participants were randomly allocated to the Weighted Squats, Fake Stimulation, and Real Stimulation groups using a computer-generated random number sequence, with 12 participants in each group. The random allocation sequence was generated by a researcher who was not involved in data collection, intervention delivery, or data analysis. Allocation concealment was maintained using sequentially numbered, opaque, sealed envelopes, which were opened only after baseline measurements had been completed.

Participants in the Fake Stimulation and Real Stimulation groups were blinded to stimulation condition because both conditions used identical electrode placement, device connection, and operating procedures. In the Fake Stimulation group, the stimulation intensity was maintained at or below the sensory threshold and did not induce visible muscle contraction. Complete participant blinding across all three groups was not possible because the Weighted Squats group did not receive electrode placement. However, all participants were blinded to the study hypotheses and expected group effects. Assessors responsible for timing, video-based movement-cycle identification, EMG data processing, and statistical analysis were blinded to group allocation through coded participant files.

This study used a randomized, parallel-group, acute intervention design. Participants with comparable competitive levels were randomly assigned to one of three groups: Weighted Squats, Fake Stimulation, or Real Stimulation. Each participant completed one assigned intervention condition, followed by the Forward Lunge footwork performance test. The aim was to compare the acute effects of three pre-activation strategies on Forward Lunge performance and neuromuscular control variables.

The experiment was conducted in an indoor testing area. The star-shuttle test was arranged with a central starting point and eight target points placed in the anterior, posterior, left, right, and four diagonal directions. The distance from the center to each target point was 3 m. Participants moved from the center to each target point in a prescribed order, touched the target point, and returned to the center until all directions were completed. The Forward Lunge task was defined as the anterior lunge movement from the center to the forward target point. Timing gates were positioned at the start–finish line to record the total completion time.

All participants completed a standardized warm-up, MVC testing for the 14 target muscles, the assigned acute intervention, and six valid star-shuttle trials with synchronized surface electromyography and high-speed video recording. A 60–90 s rest interval was provided between trials. Trials were repeated if premature start, incorrect route, missed target contact, loss of balance, or recording errors occurred. One standard Forward Lunge cycle was extracted from each valid trial for neuromuscular analysis.

The Weighted Squats group performed a conventional pre-activation protocol consisting of weighted squats only. The load was set at 60% of one-repetition maximum, and participants completed 3 sets of 5 repetitions with 2 min of rest between sets (Wilson et al., 2013).

The Fake Stimulation group performed the same weighted squat protocol with Fake Stimulation. Electrode placement and device procedures were identical to those used in the Real Stimulation group, but stimulation intensity was maintained at or below the sensory threshold and did not induce visible muscle contraction.

The Real Stimulation group performed the same weighted squat protocol with effective neuromuscular electrical stimulation applied bilaterally over the quadriceps femoris. Stimulation frequency was 75 Hz, pulse width was 400 μs, and the duty cycle was 6 s:12 s. Stimulation intensity was adjusted to 80%–90% of the individual maximal tolerable intensity to induce visible and tolerable muscle contractions.

The performance outcome was the total completion time of the star-shuttle test. Neuromuscular variables were calculated from the Forward Lunge cycle extracted from each valid trial, including iEMG, co-activation index, and intermuscular time-frequency coherence. Each participant’s final value for each variable was calculated as the mean of six valid trials.

### 2.4 Selection of target muscles

Forward Lunge is a typical movement pattern in the badminton star-shuttle test. The movement includes initiation, lunging, landing and braking, support stabilization, and push-off recovery. Although this movement is primarily characterized by rapid lower-limb displacement, it is not generated by the lower limbs alone; rather, it requires coordinated involvement of the trunk, upper limbs, and lower limbs. The lower-limb muscles mainly contribute to initiation, lunging, braking, and push-off; the trunk muscles contribute to postural stability, center-of-mass control, and force transmission; and the upper-limb muscles assist in maintaining balance and sport-specific movement posture during rapid displacement (Huang et al., 2019).

All participants were right-side dominant. To minimize the influence of side dominance on electromyographic signals and to improve comparability across participants, surface electromyographic signals were recorded from the right side only. Based on the biomechanical characteristics of the Forward Lunge and the requirements of intermuscular time-frequency coherence analysis, 14 right-side target muscles were selected: gluteus maximus (GLM), vastus medialis (VM), vastus lateralis (VL), biceps femoris (BF), medial gastrocnemius (GM), lateral gastrocnemius (GL), rectus abdominis (ABS), latissimus dorsi (LD), trapezius (TRAP), pectoralis major (PM), deltoid (DEL), biceps brachii (BB), triceps brachii (TB), and brachioradialis (BRD).

### 2.5 Forward lunge movement cycle definition

The Forward Lunge is a representative badminton-specific footwork pattern within the star-shuttle test. It involves movement initiation, lunging, landing and braking, support stabilization, and push-off recovery. In this study, the Forward Lunge direction in the star-shuttle test was selected as the target movement for analyzing footwork performance and neuromuscular control (Phomsoupha and Laffaye, 2015).

The Forward Lunge cycle was identified offline from high-speed video recordings using frame-by-frame analysis. One complete cycle was defined as the period from movement initiation at the central starting position to completion of the lunge, return to the center, and re-establishment of a stable stance. The cycle onset was defined as the first frame in which the participant-initiated movement toward the Forward Lunge direction, indicated by initial lift-off of the stepping limb or clear forward displacement of the body center of mass. The cycle offset was defined as the first frame in which the participant returned to the central position and regained stable bilateral support.

The Forward Lunge cycle was divided into three phases: the initiation phase, from the central ready position to stepping-limb lift-off or clear forward displacement of the body center of mass; the lunge-support phase, from stepping-limb lift-off to foot contact at the target area and completion of braking support; and the push-off recovery phase, from the end of braking support to return to the central position with stable stance. Two trained researchers independently identified the cycle onset, foot-contact event, and cycle offset from the high-speed video recordings. Disagreements were resolved by a third researcher. The identified cycle was then aligned with the surface electromyography signals using the synchronization trigger, and the corresponding EMG segment was extracted and time-normalized for subsequent neuromuscular analyses. The testing environment is shown in **Figure 1**.

### 2.6 Data processing and analysis

#### 2.6.1 Performance data processing

The total completion time of the star-shuttle test was automatically recorded and exported using the Microgate Witty Timing System. Before analysis, all trials were visually screened for validity. Trials were excluded and repeated after sufficient rest if a premature start, incorrect movement route, missed target contact, loss of balance, timing-gate failure, or recording error occurred.

Each participant completed six valid star-shuttle trials after the assigned intervention. The mean completion time of the six valid trials was used as the final performance outcome for each participant. The total completion time of the star-shuttle test was used as the primary performance variable, with a shorter completion time indicating better Forward Lunge footwork performance.

#### 2.6.2 Surface EMG signal preprocessing

Surface EMG data were processed using MATLAB and Python. Raw EMG signals were first inspected for signal quality. Trials with electrode detachment, signal saturation, severe motion artefacts, or synchronization errors were excluded from further analysis.

For each valid trial, one standard Forward Lunge movement cycle was identified from the high-speed video recordings, and the corresponding EMG segment from the 14 target muscles was extracted. Raw EMG signals were demeaned to remove DC offset and then filtered using a fourth-order zero-phase Butterworth band-pass filter with a cut-off frequency range of 20–400 Hz. The filtered signals were full-wave rectified.

Each Forward Lunge cycle was time-normalized to 500 data points using linear interpolation. The EMG envelope used for iEMG and co-activation index analysis was obtained from the rectified and smoothed EMG signal. Intermuscular time-frequency coherence analysis was performed using the demeaned and band-pass filtered EMG signals to preserve the frequency-domain information. Time-frequency coherence analysis was performed using the demeaned and band-pass filtered EMG signals to preserve the frequency-domain information.

Before statistical analysis, all EMG trials were visually and quantitatively inspected for signal quality. Trials were excluded if they met any of the following criteria: electrode detachment or obvious electrode displacement during movement; signal saturation or clipping; excessive motion artefact during the Forward Lunge cycle; abnormal baseline drift; missing synchronization trigger; incomplete video identification of the movement cycle; or signal amplitude exceeding three standard deviations from the participant’s mean value for the same muscle across repeated trials. Trials with incorrect movement route, missed target contact, premature start, or loss of balance were also excluded and repeated after sufficient rest.

Each participant was required to complete six valid trials. When a trial was excluded during data collection, an additional trial was recorded until six valid trials were obtained. The number of excluded trials was recorded for each participant and compared among groups to determine whether signal quality differed systematically across intervention conditions.

To assess the reliability of EMG-derived variables across the six valid trials, intraclass correlation coefficients were calculated using a two-way mixed-effects model with absolute agreement. Coefficients of variation were also calculated for the primary EMG-derived variables. Reliability was interpreted using standard criteria, with ICC values greater than 0.75 indicating good reliability and values greater than 0.90 indicating excellent reliability.

#### 2.6.3 MVC normalization

Maximum voluntary contraction (MVC) was used to normalize EMG amplitude. Before the experimental trials, MVC tests were performed for the 14 target muscles. Each muscle was tested three times, with each contraction lasting 3–5 s and 30–60 s of rest between trials.

MVC signals were processed using the same preprocessing procedures as the experimental EMG data, including demeaning, band-pass filtering, and full-wave rectification. For each muscle, the maximum EMG amplitude obtained across the three MVC trials was used as the reference value. EMG signals recorded during the Forward Lunge cycle were normalized to the corresponding MVC value and expressed as %MVC (Hermens et al., 2000):


$$EMG_{norm}\left(t\right)=\frac{EMG\left(t\right)}{EMG_{MVC}}\times 100\%$$

where *EMG_norm_*(*t*) is the normalized EMG amplitude, *EMG*(*t*) is the EMG amplitude at time *t* during the Forward Lunge cycle, and *EMG_MVC_* is the maximum EMG amplitude obtained during the MVC test for the corresponding muscle.

MVC testing was used as a standardization procedure to check the relative activation level of each muscle and to improve comparability of EMG amplitude across participants and muscles. However, the iEMG values reported in the Results were calculated from the processed EMG amplitude signals before MVC percentage conversion and are therefore expressed in μV·s.

#### 2.6.4 Muscle activation analysis

Integrated electromyography (iEMG) was calculated to quantify the overall muscle activation level during the Forward Lunge movement cycle. iEMG was calculated from the normalized EMG signal for each target muscle:


$$iEMG=\sum_{k=1}^{N}|EMG_{norm}\left(k\right)|\text{Δ}t$$

where *EMG_norm_*(*k*) is the normalized EMG amplitude at the *k*-th sample point, *N* is the number of samples within the Forward Lunge cycle, and Δ*t* is the sampling interval.

For each participant, iEMG was calculated for each of the six valid Forward Lunge cycles, and the mean value was used for statistical analysis.

Integrated electromyography (iEMG) was calculated to quantify the overall muscle activation level during the Forward Lunge movement cycle. iEMG was calculated from the processed EMG amplitude signal for each target muscle after demeaning, band-pass filtering, full-wave rectification, and smoothing:

where EMG is the processed EMG amplitude at the i-th sample point, N is the number of samples within the Forward Lunge cycle, and Δt is the sampling interval. Accordingly, iEMG was expressed as μV·s. MVC testing was used as a standardization procedure for EMG signal quality control and cross-participant comparability, but the reported iEMG values were retained in μV·s. For each participant, iEMG was calculated for each of the six valid Forward Lunge cycles, and the mean value was used for statistical analysis.

#### 2.6.5 Co-activation index analysis

The co-activation index (CI) was calculated to quantify the simultaneous activation of selected functional muscle pairs during the Forward Lunge cycle. CI was calculated using the normalized EMG envelopes. Based on the target muscles and the functional demands of the Forward Lunge task, the analyzed muscle pairs included VL–BF, GLM–BF, VM–BF, PM–LD, TB–BB, DEL–LD, and ABS–TRAP.

CI was calculated as:


$$CI=\frac{2\int_{t_{1}}^{t_{2}}\min[EMG_{1}\left(t\right),EMG_{2}\left(t\right)]dt}{\int_{t_{1}}^{t_{2}}[EMG_{1}\left(t\right)+EMG_{2}\left(t\right)]dt}\times 100\%$$

where *EMG*_1_(*t*) and *EMG*_2_(*t*) are the normalized EMG envelopes of the two muscles, and *t*_1_ and *t*_2_ represent the onset and offset of the Forward Lunge cycle. For each participant, CI was calculated for each of the six valid cycles, and the mean value was used for statistical analysis.

#### 2.6.6 Intermuscular time-frequency coherence analysis

Intermuscular time-frequency coherence (TFC) was used to quantify the frequency-specific synchronization between selected muscle pairs during the Forward Lunge cycle. Unlike NMF, which was based on the EMG envelope, TFC was calculated from the demeaned and band-pass filtered EMG signals to preserve spectral information (Lachaux et al., 2002).

For each selected muscle pair, short-time Fourier transform (STFT) was applied to the EMG signals, and coherence was calculated in the time-frequency domain:


$$C_{xy}\left(\tau ,f\right)=\frac{|S_{xy}\left(\tau ,f\right){|}^{2}}{S_{xx}\left(\tau ,f\right)S_{yy}\left(\tau ,f\right)}$$

where *C_xy_*(*τ*, *f*) is the coherence between muscles *x* and *y* at time window and frequency *f*, *S_xy_*(*τ*, *f*) is the cross-spectral density, and *S_xx_*(*τ*, *f*) and *S_yy_*(*τ*, *f*) are the auto-spectral densities of the two signals. Coherence values range from 0 to 1, with higher values indicating stronger synchronization at the corresponding time-frequency point.

STFT was performed using a Hamming window with a window length of 200 ms and 75% overlap. The frequency range was set to 0–50 Hz. Three frequency bands were analyzed: α band, 8–15 Hz; β band, 15–30 Hz; and γ band, 30–50 Hz. For each participant, TFC values were calculated for the six valid Forward Lunge cycles and averaged for statistical analysis.

### 2.7 Statistical analysis

All statistical analyses were performed using SPSS 26.0. Continuous variables are presented as mean ± standard deviation. Before statistical analysis, all data were checked for completeness, outliers, and distributional characteristics. Normality was assessed using the Shapiro–Wilk test, and homogeneity of variance was assessed using Levene’s test. Each participant completed six valid trials, and the mean value of the six trials was used as the final statistical value for each variable.

The primary outcome was Forward Lunge Test completion time, which reflected badminton-specific movement performance. Secondary outcomes included iEMG, co-activation index, and intermuscular time-frequency coherence in the α, β, and γ frequency bands. The sample size calculation was based on the primary outcome; therefore, the secondary neuromuscular outcomes were interpreted as supportive mechanistic outcomes.

Between-group differences were first analyzed using one-way analysis of variance. To control for multiple comparisons across the secondary neuromuscular outcomes, the Benjamini–Hochberg false discovery rate correction was applied separately within each outcome family: iEMG variables, co-activation index variables, and intermuscular time-frequency coherence variables. Statistical significance for the omnibus ANOVA results of secondary outcomes was interpreted after false discovery rate correction. When a corrected significant main effect was detected, Bonferroni-adjusted *post hoc* tests were performed to identify pairwise differences among the Weighted Squats, Fake Stimulation, and Real Stimulation groups.

If the assumptions of normality or homogeneity of variance were not met, appropriate data transformation was first applied. If the transformed data still did not meet the assumptions for parametric testing, the Kruskal–Wallis test was used for between-group comparisons, followed by appropriate non-parametric *post hoc* tests for pairwise comparisons. ANOVA results are reported as F values, uncorrected P values, and η², with statistical significance interpreted according to the corrected results for secondary outcomes. The statistical significance level was set at α = 0.05 (Lakens, 2013).

## 3 Results

### 3.1 Forward lunge test performance

Forward Lunge Test completion time was defined as the primary outcome. The iEMG, co-activation index, and intermuscular time-frequency coherence variables were defined as secondary neuromuscular outcomes. For these secondary outcomes, statistical significance was interpreted after Benjamini–Hochberg false discovery rate correction within each outcome family. The significant neuromuscular findings reported below remained significant after correction and were interpreted as supportive mechanistic outcomes.

For neuromuscular outcomes involving multiple ANOVAs, statistical significance was interpreted after Benjamini–Hochberg false discovery rate correction within each outcome family. The significant iEMG, co-activation index, and intermuscular time-frequency coherence findings reported below remained significant after false discovery rate correction.

**Figure 2:**
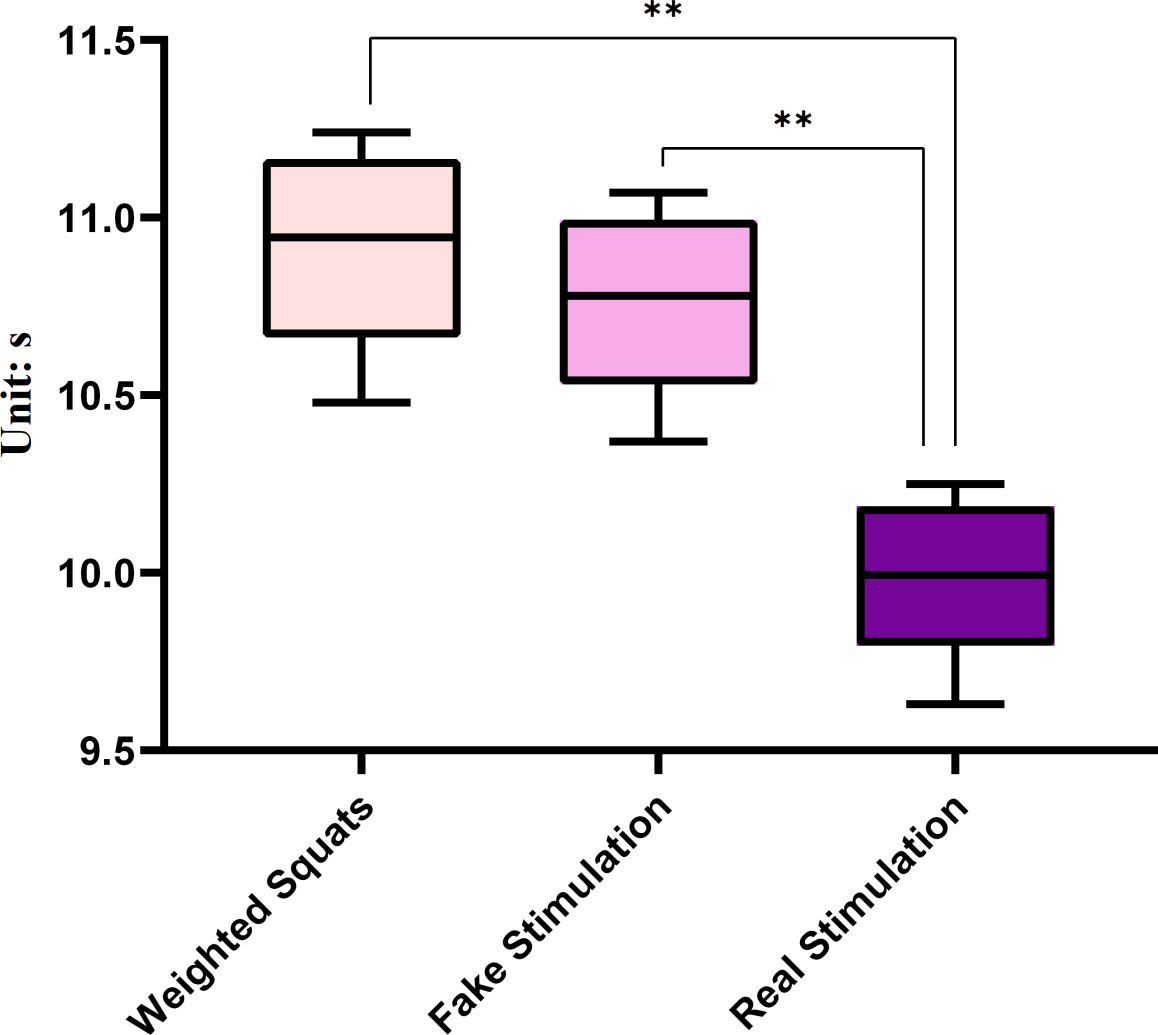
Results of the forward lunge test; ** indicates P< 0.01, indicating a statistically significant difference.

As shown in **Table 2**, **Figure 2**, Forward Lunge Test performance, assessed using the total completion time of the badminton-specific star-shuttle test, differed significantly among the three groups (F = 25.42, P< 0.001, η² = 0.61). *Post hoc* comparisons showed that the Real Stimulation group had a significantly shorter completion time than both the Weighted Squats group and the Fake Stimulation group (P< 0.05). No significant difference was observed between the Weighted Squats and Fake Stimulation groups (P > 0.05). These results indicate that Real Stimulation improved the completion time of the badminton-specific movement task that included the Forward Lunge component.

**Table 2:** Results of NMES on forward lunge performance (M ± SD, Unit: s).

Variable	Weighted squats	Fake stimulation	Real stimulation	F-value	P-value	η²
Total time (s)	10.86 ± 0.38	10.72 ± 0.35	9.94 ± 0.31bc	25.42	<0.001	0.61

b indicates a significant difference between weighted squats and the real stimulus; c indicates a significant difference between the sham stimulus and the real stimulus. P< 0.05 indicates a significant difference.

### 3.2 Muscle activation levels

**Figure 3:**
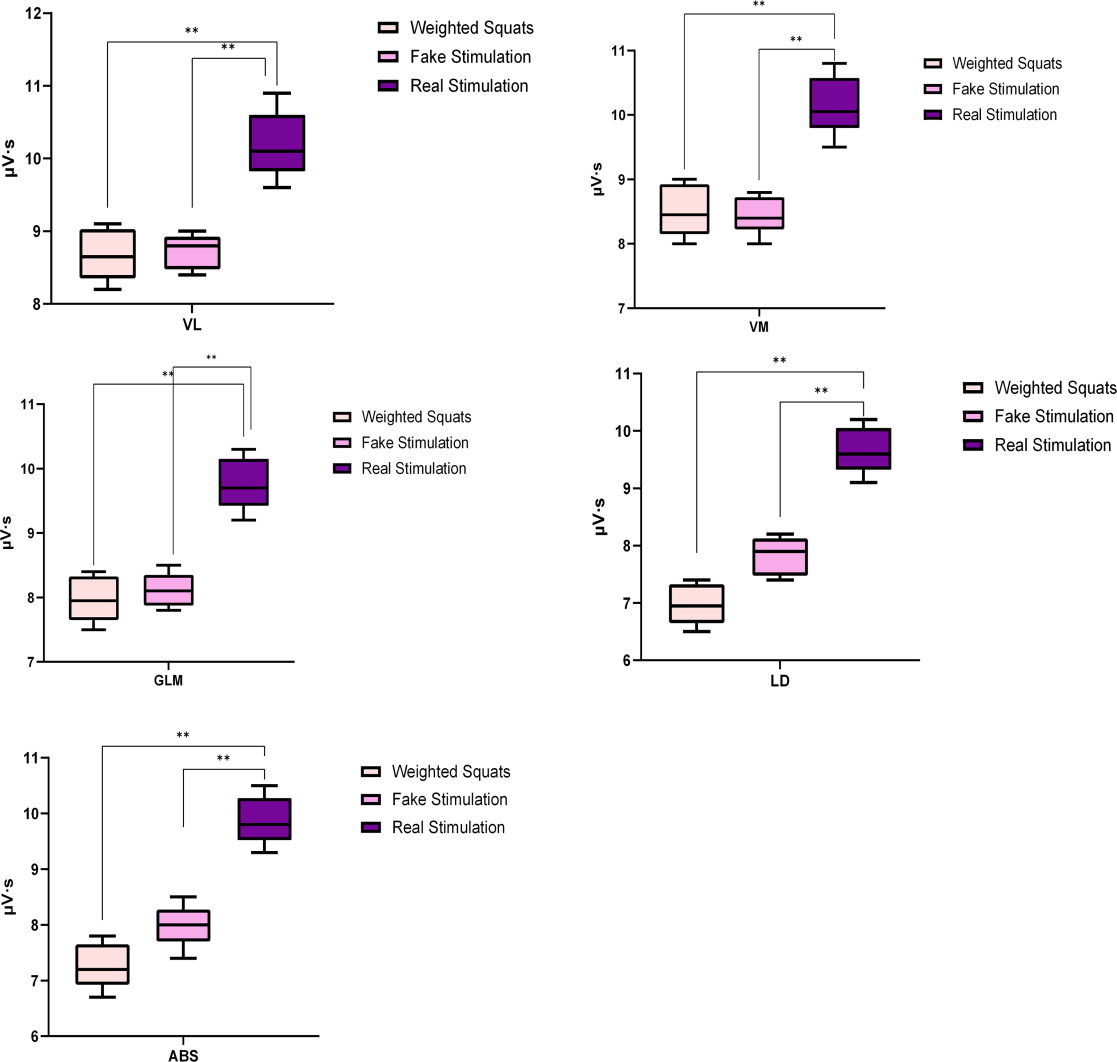
iEMG results following NMES intervention; ** indicates P< 0.01, indicating a statistically significant difference.

As shown in **Table 3**, **Figure 3**, during the Forward Lunge movement cycle, significant between-group differences in iEMG were observed for GLM, VL, VM, LD, and ABS. Specifically, GLM iEMG differed significantly among the three groups, with the Real Stimulation group showing significantly higher values than both the Weighted Squats and Fake Stimulation groups (P< 0.05). Similar significant group effects were observed for VL, VM, LD, and ABS, with the Real Stimulation group showing significantly higher iEMG values than the Weighted Squats and Fake Stimulation groups in each of these muscles (P< 0.05). No significant between-group differences were observed for BF, GM, GL, PM, TRAP, TB, BB, DEL, or BRD (P > 0.05).

**Table 3:** Results of processed muscle iEMG following NMES intervention (M ± SD, Unit: μV·s).

Muscle	Weighted squats	Fake stimulation	Real stimulation	F-value	P-value	η²
GLM	7.5 ± 1.6	7.8 ± 1.8	9.8 ± 2.4bc	18.24	<0.001	0.52
VL	8.3 ± 1.5	8.6 ± 1.7	9.8 ± 2.4bc	20.15	<0.001	0.55
VM	7.8 ± 1.8	8.2 ± 1.9	9.8 ± 2.4bc	7.56	0.001	0.35
BF	7.4 ± 1.8	7.9 ± 2.0	8.3 ± 2.3	2.12	0.132	0.12
GM	7.5 ± 1.8	8.0 ± 1.9	8.4 ± 2.1	1.87	0.167	0.10
GL	7.2 ± 1.8	7.7 ± 1.9	8.0 ± 2.2	1.56	0.221	0.08
PM	7.2 ± 1.7	8.2 ± 1.9	9.2 ± 2.2	2.34	0.056	0.15
ABS	7.0 ± 1.5	7.8 ± 1.7	9.6 ± 2.2bc	21.56	<0.001	0.58
LD	6.8 ± 1.3	7.5 ± 1.6	9.4 ± 2.1bc	24.38	<0.001	0.62
TRAP	6.5 ± 1.8	6.0 ± 1.9	5.2 ± 1.9	2.18	0.075	0.12
TB	5.8 ± 1.7	7.2 ± 1.8	8.4 ± 2.6	2.56	0.071	0.18
BB	5.6 ± 1.8	5.2 ± 1.9	4.5 ± 2.5	2.14	0.088	0.11
DEL	5.6 ± 1.8	5.9 ± 1.9	6.1 ± 3.0	0.52	0.598	0.03
BRD	4.8 ± 1.6	5.2 ± 1.7	5.5 ± 2.8	1.45	0.246	0.07

b indicates a significant difference between weighted squats and the real stimulus; c indicates a significant difference between the sham stimulus and the real stimulus. P< 0.05 indicates a significant difference. GLM, gluteus maximus; VM, vastus medialis; VL, vastus lateralis; BF, biceps femoris; GM, medial gastrocnemius; GL, lateral gastrocnemius; ABS, rectus abdominis; LD, latissimus dorsi; TRAP, trapezius; PM, pectoralis major; DEL, deltoid; BB, biceps brachii; TB, triceps brachii; BRD, brachioradialis. Statistical significance for iEMG outcomes was interpreted after Benjamini–Hochberg false discovery rate correction across the iEMG variables.

**Figure 4:**
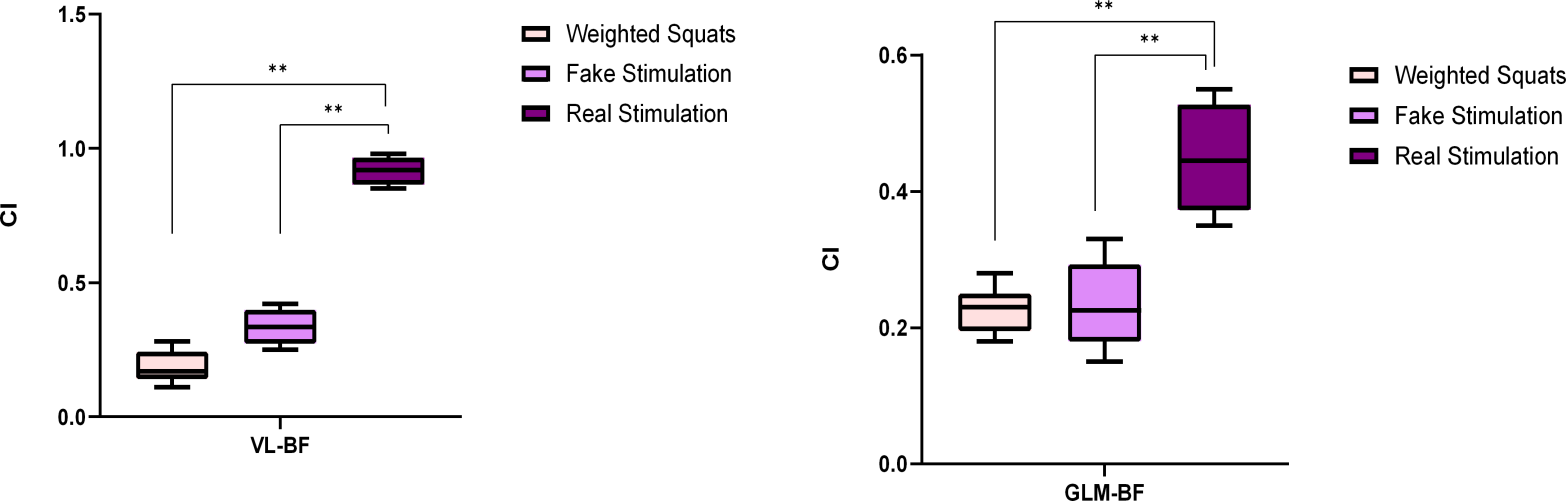
Results for co-activation levels; ** indicates P< 0.01, indicating a statistically significant difference.

As shown in **Table 4**, **Figure 4**, the co-activation index of VL–BF differed significantly among the three groups (F = 62.38, P< 0.001, η² = 0.82). *Post hoc* comparisons showed that the Real Stimulation group had a significantly higher VL–BF co-activation index than the Weighted Squats and Fake Stimulation groups (P< 0.05), whereas no significant difference was observed between the Weighted Squats and Fake Stimulation groups (P > 0.05). The co-activation index of GLM–BF also differed significantly among the three groups (F = 14.56, P< 0.001, η² = 0.48), with higher values in the Real Stimulation group than in the other two groups (P< 0.05). No significant between-group differences were observed for VM–2BF, PM–LD, TB–BB, DEL–LD, or ABS–TRAP (P > 0.05). These findings indicate that Real Stimulation increased selected lower-limb muscle-pair co-activation during the Forward Lunge movement cycle.

**Table 4:** Results of the muscle co-activation index for the forward lunge following NMES intervention.

Muscle pairs	Weighted squats	Fake stimulation	Real stimulation	F-value	P-value	η²
VL-BF	0.22 ± 0.06	0.25 ± 0.07	0.92 ± 0.06bc	62.38	<0.001	0.82
GLM-BF	0.15 ± 0.06	0.18 ± 0.07	0.45 ± 0.10bc	14.56	<0.001	0.48
VM-BF	0.52 ± 0.10	0.57 ± 0.11	0.64 ± 0.05	2.88	0.062	0.18
PM-LD	0.20 ± 0.05	0.25 ± 0.06	0.37 ± 0.09	2.64	0.168	0.15
TB-BB	0.32 ± 0.10	0.37 ± 0.09	0.38 ± 0.04	2.52	0.571	0.14
DEL-LD	0.49 ± 0.10	0.46 ± 0.10	0.45 ± 0.10	0.75	0.478	0.04
ABS-TRAP	0.39 ± 0.10	0.42 ± 0.11	0.43 ± 0.11	0.68	0.513	0.04

b indicates a significant difference between weighted squats and the real stimulus; c indicates a significant difference between the sham stimulus and the real stimulus. P< 0.05 indicates a significant difference. GLM, gluteus maximus; VM, vastus medialis; VL, vastus lateralis; BF, biceps femoris; GM, medial gastrocnemius; GL, lateral gastrocnemius; ABS, rectus abdominis; LD, latissimus dorsi; TRAP, trapezius; PM, pectoralis major; DEL, deltoid; BB, biceps brachii; TB, triceps brachii; BRD, brachioradialis. Statistical significance for co-activation outcomes was interpreted after Benjamini–Hochberg false discovery rate correction across the co-activation index variables.

### 3.3 Intermuscular time-frequency coherence

**Figure 5:**
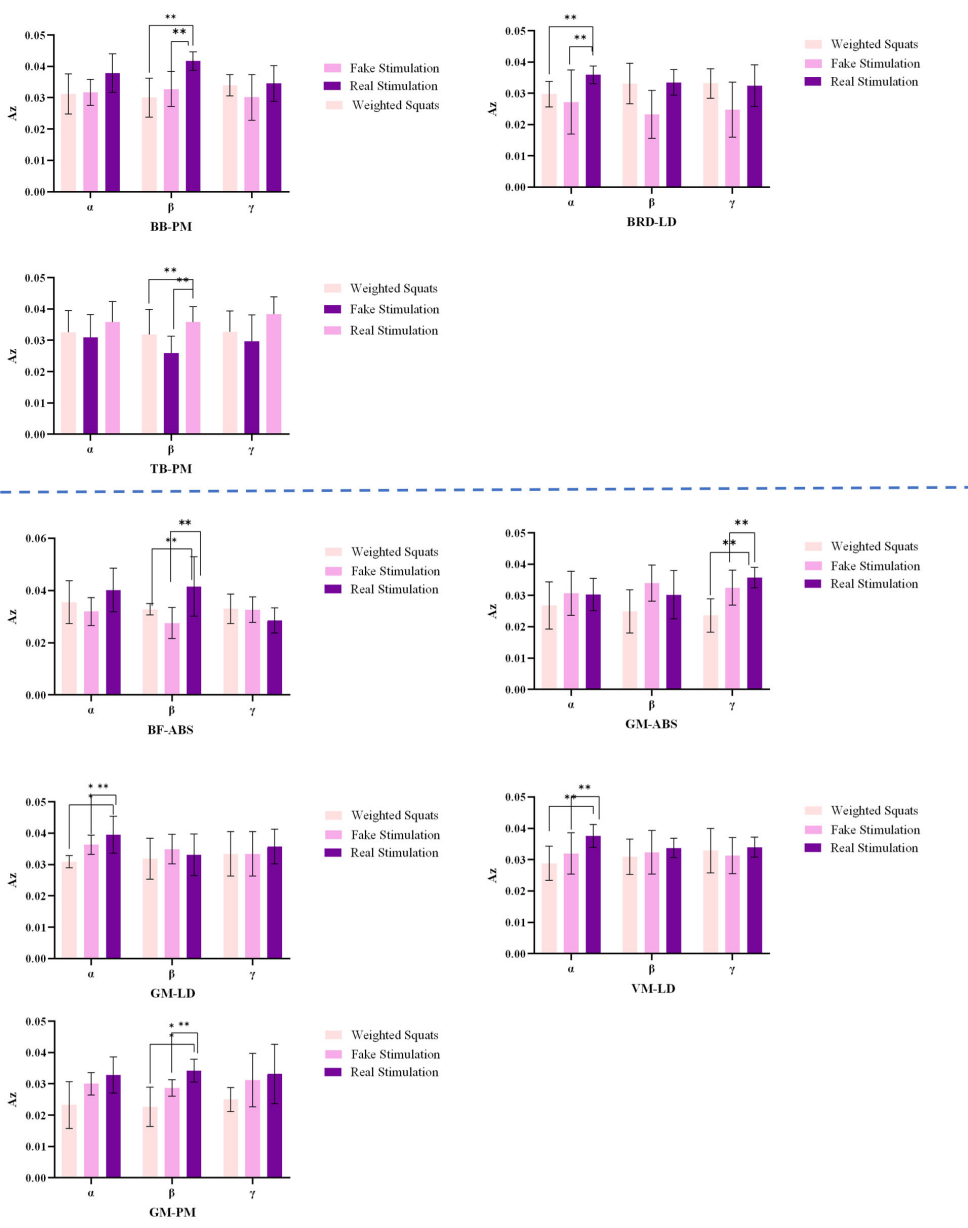
The figure shows group comparisons of intermuscular coherence for selected muscle pairs across the α, β, and γ frequency bands. The three groups are labeled as Weighted Squats, Fake Stimulation, and Real Stimulation. The α band was defined as 8–15 Hz, the β band as 15–30 Hz, and the γ band as 30–50 Hz. Significant between-group differences are indicated by asterisks. Higher coherence values indicate stronger frequency-specific synchronization between the corresponding muscle pairs during the Forward Lunge movement cycle. **P< 0.01 indicates a statistically significant difference.

**Figure 6:**
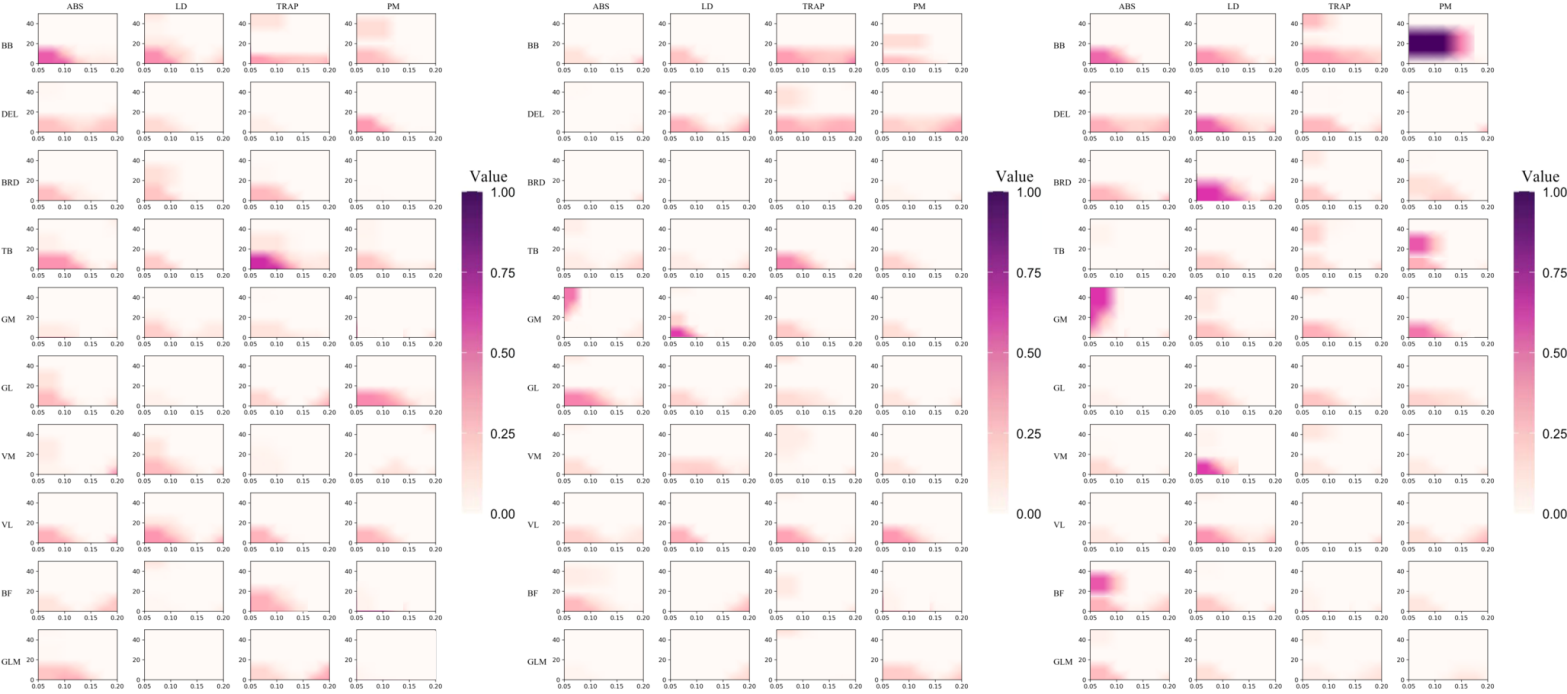
The heatmaps show time-frequency coherence between selected muscle pairs during the Forward Lunge movement cycle. The horizontal axis represents normalized movement time, and the vertical axis represents frequency. The α, β, and γ frequency bands correspond to 8–15 Hz, 15–30 Hz, and 30–50 Hz, respectively. Color intensity represents the magnitude of coherence, with warmer/darker colors indicating stronger intermuscular coupling. The group labels indicate Weighted Squats, Fake Stimulation, and Real Stimulation. Regions with stronger coherence in the Real Stimulation group indicate enhanced task-specific neuromuscular coupling during the Forward Lunge movement cycle.

As shown in **Table 5**; **Figures 5**, **6**, significant between-group differences in intermuscular time-frequency coherence were observed for selected muscle pairs and frequency bands during the Forward Lunge movement cycle. Specifically, the Real Stimulation group showed significantly higher β-band coherence than the Weighted Squats and Fake Stimulation groups for BB–PM, TB–PM, BF–ABS, and GM–PM (P< 0.05). In the α band, significantly higher coherence was observed in the Real Stimulation group for BRD–LD, GM–LD, and VM–LD (P< 0.05). In the γ band, GM–ABS coherence was significantly higher in the Real Stimulation group than in the other two groups (P< 0.05). No significant between-group differences were observed for the remaining muscle pairs or frequency bands (P > 0.05). These results indicate that Real Stimulation selectively enhanced frequency-specific intermuscular coupling during the Forward Lunge movement cycle.

**Table 5:** Intermuscular time-frequency coherence results after NMES intervention.

Muscle pairs	frequency band	Weighted squats	Fake stimulation	Real stimulation	F-value	P-value	η²
BB-PM	α	0.032 ± 0.01	0.034 ± 0.01	0.040 ± 0.01	1.67	0.20	0.055
	β	0.034 ± 0.01	0.032 ± 0.01	0.042 ± 0.01bc	5.85	0.003	0.170
	γ	0.033 ± 0.01	0.034 ± 0.01	0.033 ± 0.01	0.83	0.44	0.028
BRD-LD	α	0.033 ± 0.01	0.026 ± 0.01	0.038 ± 0.01bc	4.95	0.001	0.148
	β	0.032 ± 0.01	0.033 ± 0.01	0.036 ± 0.01	1.44	0.25	0.048
	γ	0.025 ± 0.01	0.032 ± 0.01	0.036 ± 0.01	1.62	0.21	0.054
TB-PM	α	0.029 ± 0.01	0.031 ± 0.01	0.036 ± 0.01	1.38	0.27	0.046
	β	0.031 ± 0.01	0.028 ± 0.01	0.036 ± 0.01bc	4.60	0.004	0.139
	γ	0.032 ± 0.01	0.034 ± 0.01	0.035 ± 0.01	0.70	0.51	0.024
BF-ABS	α	0.034 ± 0.01	0.036 ± 0.02	0.039 ± 0.01	1.24	0.30	0.042
	β	0.032 ± 0.01	0.029 ± 0.01	0.038 ± 0.01bc	5.85	0.001	0.170
	γ	0.031 ± 0.01	0.034 ± 0.01	0.036 ± 0.01	1.08	0.35	0.037
GM-ABS	α	0.034 ± 0.01	0.037 ± 0.01	0.041 ± 0.01	1.60	0.22	0.053
	β	0.035 ± 0.01	0.038 ± 0.01	0.040 ± 0.01	1.25	0.30	0.042
	γ	0.025 ± 0.01	0.026 ± 0.02	0.033 ± 0.01bc	5.10	0.003	0.152
GM-LD	α	0.031 ± 0.01	0.033 ± 0.01	0.039 ± 0.01bc	6.50	0.005	0.186
	β	0.034 ± 0.01	0.038 ± 0.01	0.040 ± 0.01	1.20	0.31	0.040
	γ	0.032 ± 0.01	0.036 ± 0.01	0.039 ± 0.01	1.40	0.26	0.047
VM-LD	α	0.029 ± 0.01	0.034 ± 0.01	0.039 ± 0.01bc	4.80	0.003	0.144
	β	0.034 ± 0.01	0.038 ± 0.01	0.041 ± 0.01	1.55	0.23	0.052
	γ	0.035 ± 0.01	0.039 ± 0.01	0.042 ± 0.01	1.40	0.26	0.047
GM-PM	α	0.033 ± 0.01	0.036 ± 0.01	0.040 ± 0.01	1.35	0.27	0.045
	β	0.022 ± 0.01	0.026 ± 0.01	0.035 ± 0.01bc	4.95	0.003	0.148
	γ	0.030 ± 0.01	0.033 ± 0.01	0.034 ± 0.01	1.15	0.13	0.039

b indicates a significant difference between weighted squats and the real stimulus; c indicates a significant difference between the sham stimulus and the real stimulus. P< 0.05 indicates a significant difference. GLM, gluteus maximus; VM, vastus medialis; VL, vastus lateralis; BF, biceps femoris; GM, medial gastrocnemius; GL, lateral gastrocnemius; ABS, rectus abdominis; LD, latissimus dorsi; TRAP, trapezius; PM, pectoralis major; DEL, deltoid; BB, biceps brachii; TB, triceps brachii; BRD, brachioradialis. Statistical significance for intermuscular time-frequency coherence outcomes was interpreted after Benjamini–Hochberg false discovery rate correction across the tested muscle-pair and frequency-band variables.

## 4 Discussion

### 4.1 Effects of NMES on forward lunge footwork performance

The present study found a significant difference in Forward Lunge Test performance among the three pre-activation conditions, with the Real Stimulation group showing a shorter completion time than both the Weighted Squats and Fake Stimulation groups. No significant difference was observed between the Weighted Squats and Fake Stimulation groups. These findings suggest that acute NMES combined with weighted squats may provide an additional performance benefit beyond resistance-based pre-activation alone.

Forward Lunge footwork is a badminton-specific movement that requires rapid movement initiation, anterior stepping, landing and braking, support stabilization, and push-off recovery. Therefore, the shorter completion time observed in the Real Stimulation group may reflect improved acute readiness for high-speed lower-limb movement and rapid return-to-center performance. This result is consistent with previous evidence showing that appropriate pre-activation strategies can enhance subsequent explosive or rapid movement performance by increasing neuromuscular readiness and muscle contractile preparedness (Blazevich and Babault, 2019). It is also consistent with studies suggesting that NMES can increase peripheral muscle activation and support performance in tasks requiring rapid force production and dynamic stabilization (Maffiuletti, 2010; Bergquist et al., 2012).

Importantly, the lack of a significant difference between the Weighted Squats and Fake Stimulation groups suggests that the observed performance enhancement was unlikely to be explained solely by electrode placement, sensory perception, or expectancy effects. Instead, the visible and tolerable contractions induced by Real Stimulation may have contributed to the acute improvement in Forward Lunge Test completion time. Compared with studies focusing on general jumping, sprinting, or isolated strength tasks, the present study examined a badminton-specific movement task, which increases the practical relevance of the findings for sport-performance preparation (Vanderthommen and Duchateau, 2007). However, because this study used an acute parallel-group design, the results should be interpreted as short-term performance responses rather than evidence of long-term training adaptation.

### 4.2 Effects of NMES on muscle activation and co-activation during forward lunge footwork

The present study found significant between-group differences in iEMG for several muscles during the Forward Lunge movement cycle. Specifically, the Real Stimulation group showed higher activation in GLM, VL, VM, LD, and ABS than the Weighted Squats and Fake Stimulation groups. These results indicate that acute NMES combined with weighted squats increased the activation of selected muscles involved in lower-limb support, knee control, trunk stabilization, and upper-body postural regulation during the Forward Lunge task.

Increased activation of GLM, VL, and VM may contribute to hip and knee control during lunging, landing, and push-off recovery. The increased activation of ABS and LD may reflect greater trunk and upper-body involvement during rapid forward displacement and return-to-center movement. These findings are consistent with previous studies showing that badminton lunge movements require coordinated lower-limb and trunk muscle activity to control body position, stabilize the center of mass, and support efficient movement recovery (Kellis, 1998; Rudolph et al., 2000). They are also consistent with evidence that NMES may acutely increase muscle activation and motor unit recruitment when applied with appropriate stimulation parameters (Baratta et al., 1988).

However, the present results should not be interpreted as a generalized increase in whole-body muscle activation. Several muscles did not show significant between-group differences, suggesting that the effect of Real Stimulation was selective rather than global. This selective activation pattern is relevant because the stimulation was applied to the quadriceps region, while the observed changes involved both directly related lower-limb muscles and functionally associated trunk muscles. Thus, the iEMG findings provide supportive evidence that Real Stimulation altered task-related muscle activation during Forward Lunge footwork.

The present study also found higher VL–BF and GLM–BF co-activation indices in the Real Stimulation group than in the Weighted Squats and Fake Stimulation groups. These findings indicate that effective NMES combined with weighted squats altered selected lower-limb muscle-pair activation patterns during the Forward Lunge movement cycle. Because VL–BF and GLM–BF are functionally related to knee and hip control, increased co-activation may contribute to greater joint stabilization during landing, braking, and push-off recovery. In a high-speed badminton lunge, such stabilization may help support body control during rapid deceleration and return movement (d'Avella et al., 2003; Farina et al., 2004; Cheung et al., 2005).

Nevertheless, higher co-activation should not automatically be interpreted as better coordination or greater movement efficiency. Increased agonist–antagonist co-activation may also reflect increased joint stiffness, compensatory control, or reduced neuromuscular efficiency, depending on the movement phase and task demands. Because the present study calculated co-activation over the entire Forward Lunge movement cycle, it was not possible to determine whether the increased VL–BF and GLM–BF co-activation occurred mainly during the braking-support phase, where greater stabilization may be beneficial, or during other phases, where excessive co-activation could reduce movement efficiency. Therefore, the co-activation findings should be interpreted as evidence of altered lower-limb neuromuscular control rather than definitive evidence of improved coordination.

### 4.3 Effects of NMES on intermuscular time–frequency coherence during forward lunge footwork

The present study found that intermuscular time–frequency coherence differed significantly among the three pre-activation conditions in selected muscle pairs and frequency bands. The Real Stimulation group showed higher coherence in BB–PM, TB–PM, BF–ABS, and GM–PM in the β band; BRD–LD, GM–LD, and VM–LD in the α band; and GM–ABS in the γ band. These results suggest that acute NMES combined with weighted squats altered frequency-specific intermuscular coupling during the Forward Lunge movement cycle.

Functionally, the increased α-band coherence in BRD–LD, GM–LD, and VM–LD may indicate altered synchronization between limb and trunk-related muscles during postural control and movement stabilization. The increased β-band coherence in BB–PM, TB–PM, BF–ABS, and GM–PM may reflect stronger task-related coupling among muscles involved in upper-body posture, trunk control, lower-limb braking, and whole-body movement organization. The increased γ-band coherence in GM–ABS may suggest altered high-frequency coupling during rapid movement control. These findings are consistent with previous research indicating that intermuscular coherence can reflect common oscillatory components and functional synchronization between muscles during motor tasks (Farmer, 1998; Kenville et al., 2020).

However, the coherence results should be interpreted cautiously. Surface EMG-based coherence provides indirect information about the temporal and frequency-specific synchronization of muscle activity, but it cannot directly identify the neural sources underlying the observed coupling. Therefore, the present findings should not be taken as definitive evidence of corticospinal drive, central neural regulation, or direct neural causality. Instead, they should be interpreted as indirect evidence that Real Stimulation was associated with altered intermuscular coupling during badminton-specific Forward Lunge movement.

The selective nature of the coherence findings is also important. Significant differences were observed only in specific muscle-pair and frequency-band combinations, rather than across all tested pairs and bands. This suggests that the effect of Real Stimulation was not a nonspecific increase in coherence across the whole neuromuscular system. Instead, the results may reflect task-dependent changes in muscle coupling among lower-limb, trunk, and upper-limb muscles involved in Forward Lunge footwork (Hodges and Richardson, 1997; Kibler et al., 2006; Farina and Negro, 2015; Laine et al., 2021). These findings support the usefulness of intermuscular time–frequency coherence as an analytical tool for describing sport-specific neuromuscular coordination patterns, while also highlighting the need for caution when interpreting coherence as a neural mechanism.

Overall, the coherence findings complement the performance, iEMG, and co-activation results by showing that Real Stimulation was associated with altered frequency-specific muscle coupling during the Forward Lunge movement cycle. Nevertheless, because the study was powered for the primary performance outcome and involved multiple secondary neuromuscular comparisons, the coherence results should be viewed as supportive mechanistic evidence rather than independently powered primary endpoints.

### 4.4 Limitations

Several limitations should be acknowledged. First, the sample size was small, with only 12 participants per group, and only male badminton players were included, which limits statistical power and generalizability. Second, this study used an acute parallel-group design; therefore, the findings reflect short-term responses only and cannot determine long-term adaptations or within-participant effects across conditions. Third, although baseline characteristics were expanded, more detailed baseline strength, fatigue, and neuromuscular profiles were not collected.

Fourth, improvements in Forward Lunge Test completion time should not be interpreted as being fully explained by EMG outcomes. The iEMG, co-activation, and intermuscular coherence variables were secondary outcomes, involved multiple comparisons, and should be interpreted as supportive mechanistic evidence rather than direct causal proof. In addition, surface EMG-based coherence is an indirect measure of neuromuscular coupling and cannot identify the exact neural sources.

Finally, practical issues related to NMES should be considered, including possible discomfort, fatigue, expectancy effects, equipment availability, preparation time, athlete tolerance, and feasibility before competition. Future studies should use larger and more diverse samples, include female athletes, apply longitudinal or crossover designs, and combine EMG coherence with kinematic, kinetic, fatigue, and neurophysiological measures.

## 5 Conclusion

This study showed that acute pre-competition NMES combined with weighted squats improved Forward Lunge Test performance in male badminton players, as reflected by a shorter completion time compared with weighted squats and Fake Stimulation. This acute improvement was accompanied by higher iEMG levels in GLM, VL, VM, LD, and ABS, increased co-activation of VL–BF and GLM–BF, and stronger intermuscular time-frequency coherence in selected muscle pairs within the α, β, and γ frequency bands.

These findings suggest that NMES may serve as a useful adjunct to resistance-based pre-activation for acutely enhancing badminton-specific Forward Lunge performance. However, given the acute experimental design, male-only sample, and indirect nature of surface EMG-based coherence measures, the findings should be interpreted as short-term neuromuscular responses rather than evidence of long-term training adaptation or direct neural causality. Future studies should include female athletes, larger samples, longitudinal intervention designs, and complementary biomechanical or neurophysiological measures to further verify the mechanisms and practical application of NMES in badminton performance preparation.

## Data Availability

The original contributions presented in the study are included in the article/supplementary material. Further inquiries can be directed to the corresponding author.
